# Successful treatment of necrobiosis lipoidica and associated retinal vasculitis with tumor necrosis factor (TNF)-alpha inhibitor

**DOI:** 10.1016/j.ajoc.2023.101908

**Published:** 2023-07-26

**Authors:** Ketaki Panse, Manuela Herrera, Maxwell Fung, Alain Brassard, Kareem Moussa

**Affiliations:** a Department of Ophthalmology & Vision Science, University of California, Davis, Sacramento, CA, USA; b University of California, Davis, School of Medicine, Sacramento, CA, USA; c Department of Dermatology, University of California, Davis, Sacramento, CA, USA

**Keywords:** Occlusive retinal vasculitis, Necrobiosis lipoidica, Tumor necrosis factor-alpha inhibitor, Uveitis

## Abstract

**Purpose:**

To report the clinical and histopathologic features of necrobiosis lipoidica and associated retinal vasculitis and describe successful treatment of both skin and eye manifestations with adalimumab, a tumor necrosis factor (TNF)-alpha inhibitor.

**Observations:**

A 35-year-old patient with bipolar disorder and ocular hypertension was referred for evaluation of bilateral retinal vasculitis. Fluorescein angiography revealed bilateral occlusive retinal vasculitis. Physical exam was notable for multiple annular and round erythematous hyperpigmented and atrophic patches and plaques on both lower extremities. Skin biopsy revealed a diagnosis of necrobiosis lipoidica, a rare granulomatous skin disease. Both the patient's retinal vasculitis and skin patches responded favorably to treatment with adalimumab, a TNF-alpha inhibitor.

**Conclusions and importance:**

This case highlights the importance of obtaining a complete history and physical exam in patients who present with ocular inflammation, as extraocular manifestations of disease may be present. It also demonstrates the effectiveness of a multidisciplinary approach to evaluation and management of these patients, as both skin and eye involvement were successfully treated with adalimumab, a TNF-alpha inhibitor.

## 1 Introduction

Necrobiosis lipoidica is a rare, chronic granulomatous disease of the skin. It is a disorder that primarily affects young and middle-aged adults and is more common in women than in men. Concurrent type 1 or type 2 diabetes mellitus is common in patients with necrobiosis lipoidica with a reported prevalence of up to 65%.^1^ Limited studies suggest that there may be a correlation between the presence of necrobiosis lipoidica and the presence of diabetic complications such as renal and retinal disease.^10^

Retinal vasculitis is a vision-threatening disease and may occur in isolation or in association with a known systemic disease.^2^ In one series, approximately 55% of patients with retinal vasculitis had an associated systemic autoimmune disease.^3^ A comprehensive evaluation of patients with retinal vasculitis is necessary to minimize morbidity and mortality that may be associated with an underlying systemic disease. We present a rare case of retinal vasculitis associated with necrobiosis lipoidica successfully treated with adalimumab, a tumor necrosis factor-alpha inhibitor.

## 2 Case report

A 35-year-old woman presented to our clinic for evaluation of bilateral retinal vasculitis. Her medical history was significant for type 2 diabetes without retinopathy, bipolar disorder, and steroid-induced ocular hypertension. Family history was noncontributory.

She reported a history of peripheral vision loss and photopsias that began 3 years prior to our evaluation. She was seen by a retina specialist and treated with intravitreal corticosteroid injection and panretinal photocoagulation for occlusive retinal vasculitis. She presented to our clinic seeking a second opinion.

**Fig. 1:**
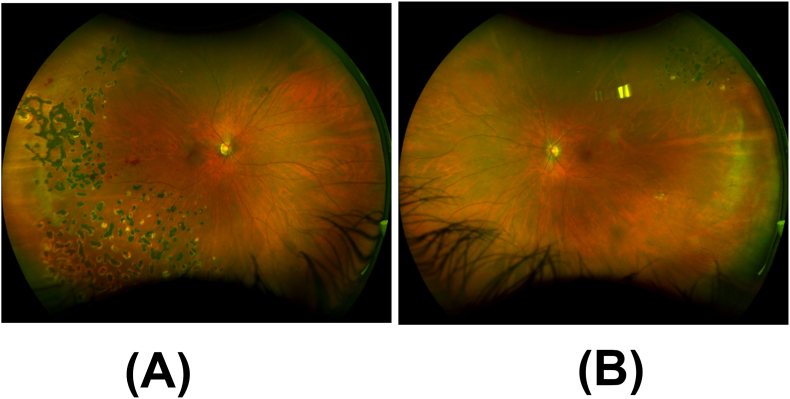
Ultra-widefield fundus image of the right eye shows (A) scattered dot blot hemorrhages, sclerotic vessels, and peripheral laser scars and (B) peripheral laser scars in the left eye.

On exam, best corrected visual acuity was 20/25 in the right eye and 20/20 in the left eye, with normal intraocular pressure in each eye on latanoprost once at bedtime in both eyes. Visual field by confrontation revealed partial outer inferotemporal, superonasal, and inferonasal deficiencies in the right eye. Slit lamp examination was notable for inflammatory cells in the anterior vitreous, scattered dot blot hemorrhages in the right retina, sclerotic vessels in both retinas, and peripheral laser scars in both retinas (Fig. 1).

**Fig. 2:**
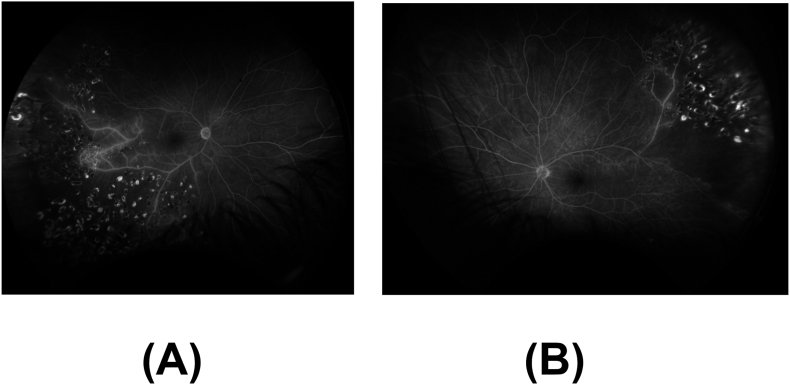
A. Fluorescein angiography (FA) of the right eye at 8:52 shows temporal non-perfusion and leakage consistent with occlusive vasculitis. B. Fluorescein angiography (FA) of the left eye at 5:20 shows large wedge capillary dropout superotemporally with mild peripheral leakage.

**Fig. 3:**
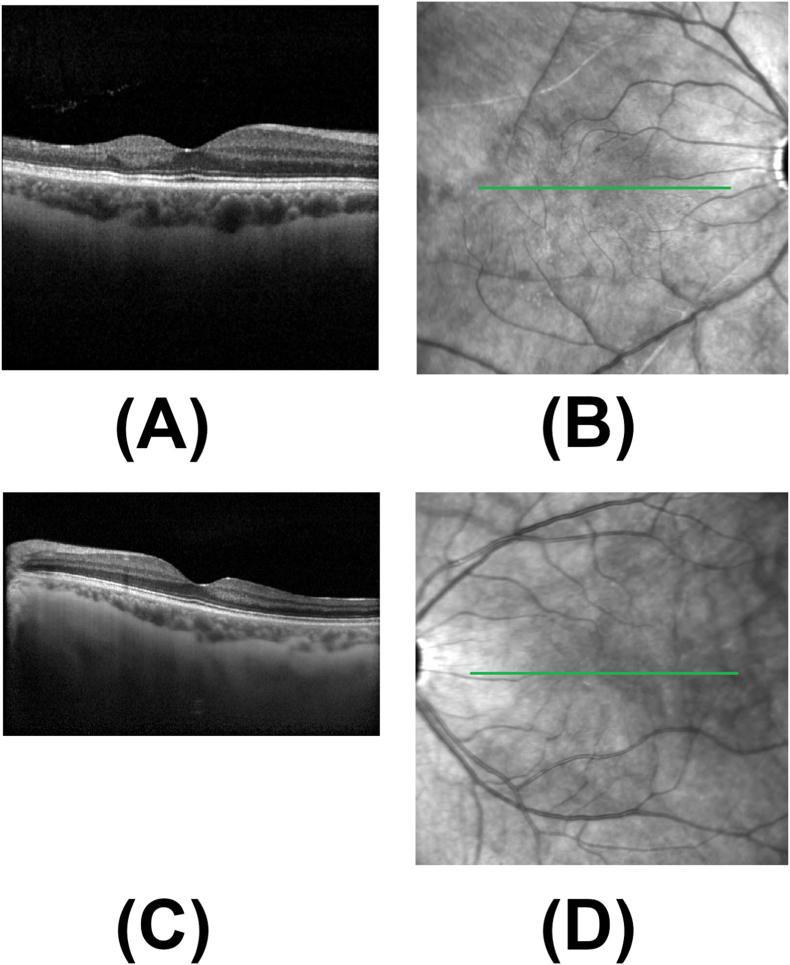
Spectral domain optical coherence tomography (SD-OCT). A. SD-OCT of the right macula shows temporal inner retinal thinning consistent with prior retinal artery occlusion. B. SD-OCT of the left macula shows temporal inner retinal thinning consistent with prior retinal artery occlusion.

Fluorescein angiography of both eyes showed temporal non-perfusion and leakage consistent with occlusive vasculitis (Fig. 2). Optical coherence tomography (OCT) of both maculae showed temporal inner retinal thinning consistent with prior retinal artery occlusions (Fig. 3).

**Fig. 4:**
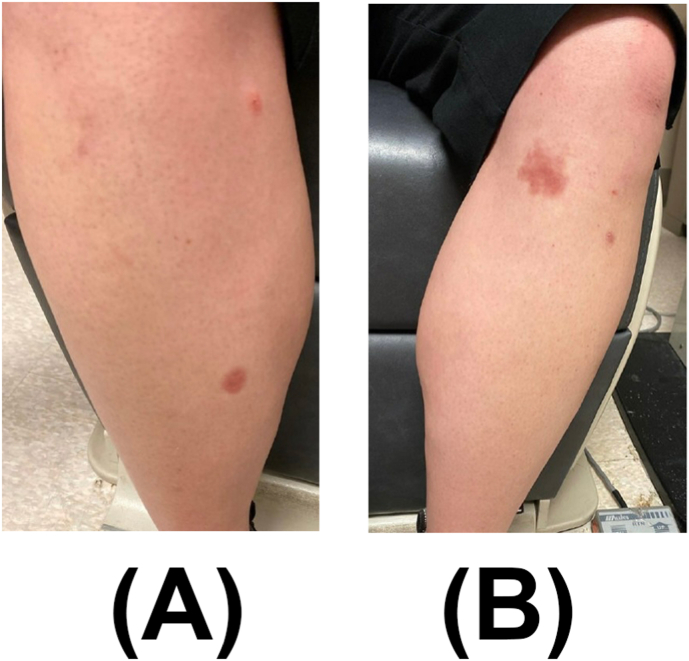
External photograph of multiple annular blanching patches on the right lower extremity (A) and left lower extremity (B).

**Fig. 5:**
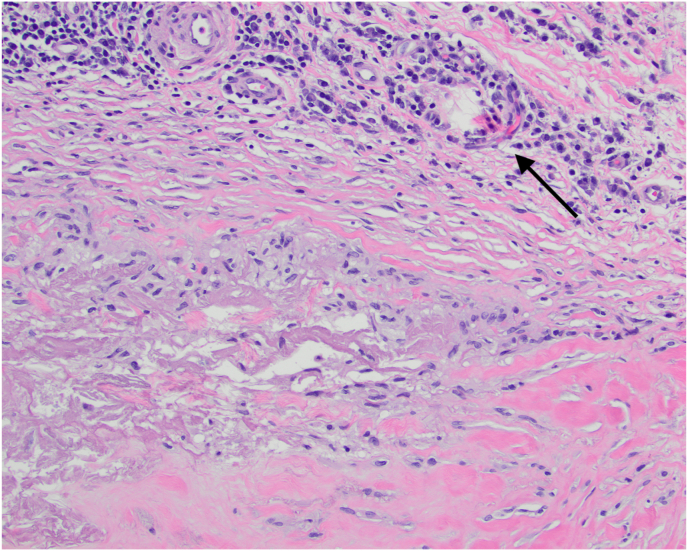
Skin biopsy showed mixed inflammatory infiltrate composed of palisaded necrobiotic histiocytes and lymphocytes including many plasma cells (black arrow) within a sclerotic reticular dermis consistent with necrobiosis lipoidica. Hematoxylin and eosin stain (H&E), x200.

Physical exam revealed multiple annular and round erythematous hyperpigmented and atrophic blanching patches and plaques on both lower extremities (Fig. 4), for which she was referred to the dermatology service. Laboratory testing for syphilis (Syphilis immunoglobulins G (IgG) and M (IgM), tuberculosis (inferferon gamma releasy assay (IGRA)), and granulomatosis with polyangiitis (antineutrophil cytoplasmic antibodies) were negative. A complete blood count was unremarkable. Chest radiography was unremarkable without signs of sarcoidosis. Biopsy of the patch on the right lower leg revealed a mixed inflammatory infiltrate composed of palisaded necrobiotic histocytes and lymphocytes including many plasma cells within a sclerotic reticular dermis, consistent with necrobiosis lipoidica (Fig. 5).

Given the patient's history of psychiatric illness and steroid-induced ocular hypertension, which may be exacerbated by systemic or local steroid, respectively, we offered the patient immunomodulatory therapy with adalimumab, which she accepted. Two months later, she developed neovascularization of the retina in the right eye, which was treated with an intravitreal bevacizumab injection and panretinal photocoagulation.

**Fig. 6:**
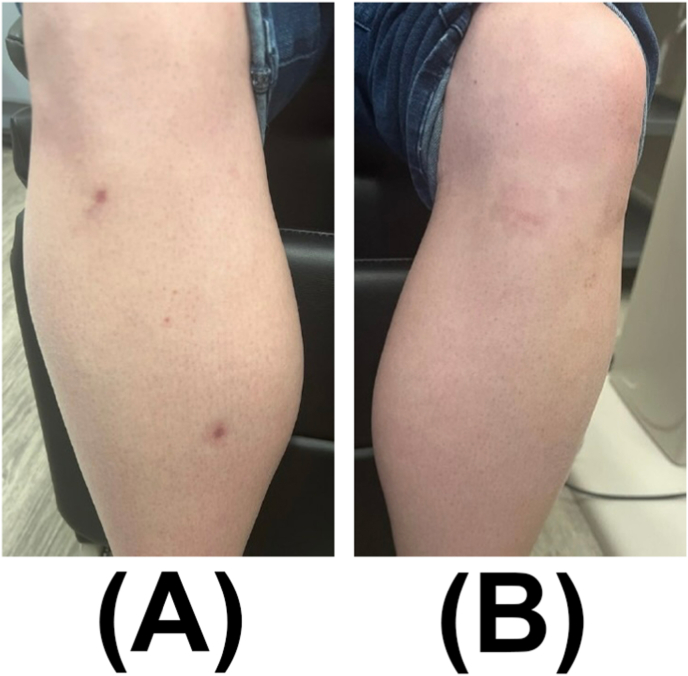
External photograph of resolving annular blanching patches on right lower extremity (A) and left lower extremity (B).

**Fig. 7:**
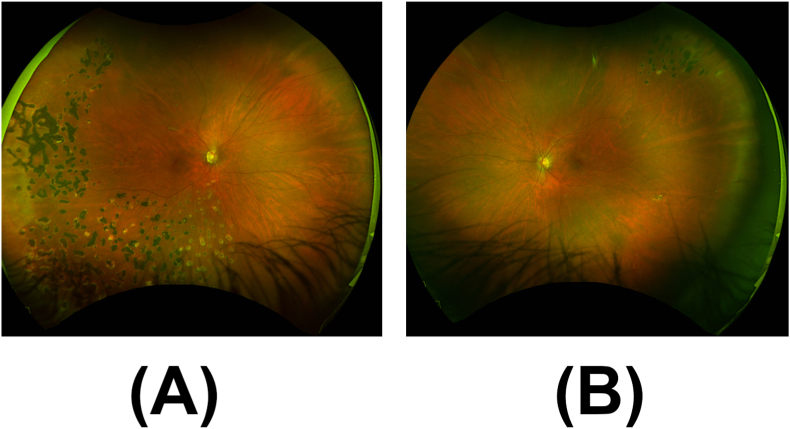
Ultra-widefield fundus image at follow up of the right eye shows (A) near complete resolution of hemorrhage and (B) peripheral laser scars in the left eye.

At last follow-up, 12 months after initial presentation, she reported no additional eye symptoms, and the skin lesions resolved (Fig. 6). BCVA was 20/20 in both eyes and IOPs were normal without IOP lowering drops. Eye exam showed regression of retinal neovascularization of the right eye, with near complete resolution of previous hemorrhages (Fig. 7).

## 3 Discussion

Necrobiosis lipoidica is a rare, chronic granulomatous disease of the skin that usually begins as red-brown papules that may develop into ulcers. The diagnosis is made based on clinical presentation and histopathology. Treatment options include topical or intralesional steroids, and/or systemic immunomodulatory therapy, including TNF-alpha inhibitors.^4^^,^^5^ Adalimumab, a TNF-alpha inhibitor, was first approved by the United States (US) Food and Drug Administration (FDA) in 2002 for use in patients with rheumatoid arthritis. It was approved for the treatment of adults with noninfectious intermediate, posterior, and panuveitis in June 2016 after a landmark clinical trial demonstrated its safety and efficacy.^7^^,^^8^

A review of the literature reveals two reports of necrobiosis lipoidica with ocular inflammation. One report from 1969 describes a 61-year-old woman with diabetes mellitus (diabetes mellitus type not reported) and sarcoidosis who developed chronic iridocyclitis in the left eye.^9^ The second report from 2011 describes a 43-year-old woman with necrobiosis lipoidica and bilateral anterior/intermediate granulomatous panuveitis with occlusive retinal vasculitis, without diabetes mellitus, that was treated with topical corticosteroid eye drops and panretinal photocoagulation for the ocular involvement, and topical and intralesional treatments for the skin lesions, with variable efficacy.^6^

To the best of our knowledge, we describe the first case of necrobiosis lipoidica and associated retinal vasculitis with favorable treatment response to adalimumab, a TNF-alpha inhibitor with demonstrated safety and efficacy in the treatment of noninfectious uveitis^.7,8^ While it is possible that the patient's retinal vasculitis and necrobiosis lipoidica may not be linked, the simultaneous occurrence of active retinal vasculitis and skin lesions, and the favorable response of both to treatment with adalimumab, suggests a unifying disease process. Ophthalmologists, dermatologists, and rheumatologists, as part of a multidisciplinary team managing patients with autoimmune disease with involvement of multiple organs, may consider the use of TNF-alpha inhibitors in the treatment of this rare disease.

## Patient consent

Written consent to publish this case has not been obtained. This report does not contain any personal identifying information.

## Acknowledgments and disclosures

None.

## Declaration of competing interest

The authors have no conflict of interest.
